# Lenalidomide Stabilizes Protein–Protein Complexes by Turning Labile Intermolecular H-Bonds into Robust Interactions

**DOI:** 10.1021/acs.jmedchem.2c01692

**Published:** 2023-04-21

**Authors:** Marina Miñarro-Lleonar, Andrea Bertran-Mostazo, Jorge Duro, Xavier Barril, Jordi Juárez-Jiménez

**Affiliations:** † Unitat de Fisicoquímica, Departament de Farmàcia i Tecnologia Farmacèutica, i Fisicoquímica, Facultat de Farmàcia i Ciències de l’Alimentació, Universitat de Barcelona (UB), Av. Joan XXIII, 27-31, 08028 Barcelona, Spain; ‡ Institut de Química Teòrica i Computacional (IQTC), Facultat de Química i Física, Universitat de Barcelona (UB), C. Martí i Franquès, 1, 08028 Barcelona, Spain; § Institut de Biomedicina, Facultat de Biologia, Universitat de Barcelona (UB), Av. Diagonal, 643, 08028 Barcelona, Spain; ∥ Catalan Institution for Research and Advanced Studies (ICREA), Pg. Lluís Companys, 23 08010 Barcelona, Spain

## Abstract

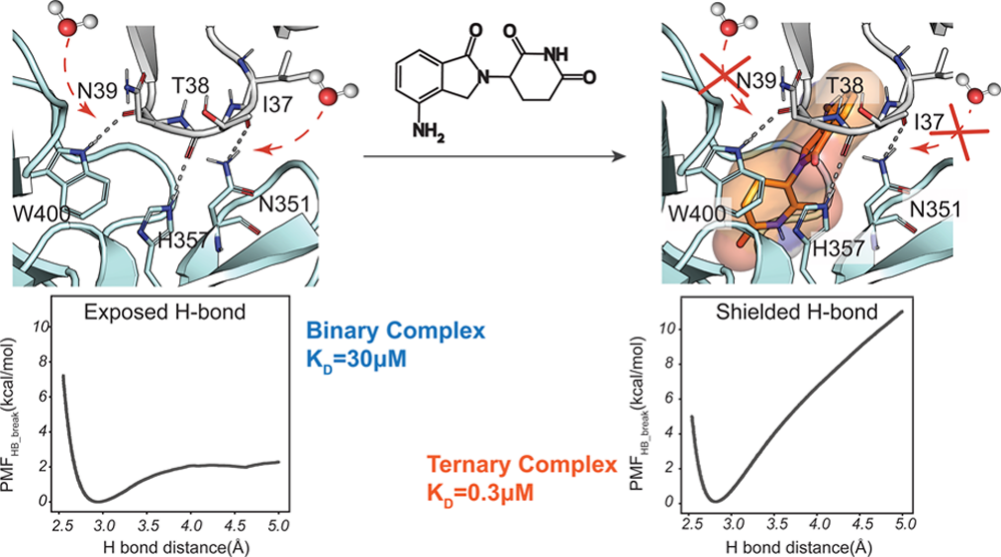

Targeted protein degradation is a promising therapeutic strategy, spearheaded by the anti-myeloma drugs lenalidomide and pomalidomide. These drugs stabilize very efficiently the complex between the E3 ligase Cereblon (CRBN) and several non-native client proteins (neo-substrates), including the transcription factors Ikaros and Aiolos and the enzyme Caseine Kinase 1α (CK1α,), resulting in their degradation. Although the structures for these complexes have been determined, there are no evident interactions that can account for the high efficiency of formation of the ternary complex. We show that lenalidomide’s stabilization of the CRBN–CK1α complex is largely due to hydrophobic shielding of intermolecular hydrogen bonds. We also find a quantitative relationship between hydrogen bond robustness and binding affinities of the ternary complexes. These results pave the way to further understand cooperativity effects in drug-induced protein–protein complexes and could help in the design of improved molecular glues and more efficient protein degraders.

## Introduction

The concept of molecular glues (MGs) was introduced by Zheng and co-workers^1^ to describe the stabilizing effect of the plant hormone auxin on several complexes with the SCF^TIR1^ ubiquitin ligase complex. Subsequently, it has been revealed that this mechanism is quite common in nature^2−5^ and that even a number of widely used drugs, such as the immunosuppressant drug Cyclosporin A^6^ or the anti-cancer agents Paclitaxel^7^ and Indisulam,^8^ share a similar mechanism of action. These findings have spurred interest in leveraging selective stabilization of protein–protein interactions in drug discovery. However, it has been repeatedly noted in the literature^9−11^ that the discovery of MGs is too reliant on serendipity and that the future development of successful MGs as therapeutic agents ought to shift into more rational approaches and further understanding of the molecular mechanisms underpinning the ligand-induced stabilization of protein–protein interactions. Contrary to traditional drug discovery, which focuses on the formation of binary complexes, rational development of MGs will require a detailed understanding of the formation of ternary complexes, which often imply non-additive mechanisms.^12^ The physicochemical factors underlying these mechanisms are usually difficult to anticipate from structural analysis or common computer-aided drug design protocols such as docking or virtual screening.^3^ Nonetheless, they are critical to the selective stabilization of protein–protein complexes and must be understood to fully exploit the therapeutic opportunities offered by MGs.

A landmark example of the potential of MGs to impact human health is provided by thalidomide derivatives lenalidomide and pomalidomide, so-called immunomodulatory drugs (IMiDs), widely used in the treatment of multiple myeloma. Only recently it was described that these molecules induce the ubiquitination and degradation of the transcription factors Ikaros (IKZF1) and Aiolos (IKZF3)^13,14^ and the enzyme Casein Kinase 1α (CK1α)^15^ by stabilizing the complex of these proteins with the E3 ligase Cereblon (CRBN),^16^ which is the substrate receptor of the CUL4–RBX1–DDB1 ubiquitin ligase complex (CRL4). IMiDs are accommodated in a tryptophan cage in the substrate binding domain of CRBN,^17^ and structural evidence has shown that IKZF1,^18^ CK1α^19^ and other proteins, collectively known as neo-substrates,^20,21^ bind to the CRBN–IMiD interface, establishing a set of protein–protein interactions through a β-hairpin loop structure that contains a glycine residue on the apex.^5,18,19^ Additionally, it was recently described that the first-ever endogenous degron moiety described (C-terminal cyclic imides) binds to the same tryptophan cage, with structural features that closely resemble those of IMiDs.^22,23^ Furthermore, another landmark study has proposed that some IMiDs are able to increase neo-substrate degradation by stabilizing a closed conformation of CRBN responsible for binding neo-substrates.^24^ In parallel, in a recent work, Cao et al.^25^ proposed that instead of creating new sets of interactions, MGs in general, and IMiDs in particular, must be able to stabilize pre-existing protein–protein interactions. They support this hypothesis by demonstrating that pomalidomide stabilizes the IKZF1–CRBN complex by around 4-fold, while lenalidomide stabilizes the CK1α–CRBN complex by around 30-fold. Analysis of the structural data available seems to support this hypothesis, as the direct intermolecular interactions between lenalidomide–CK1α^19^ and pomalidomide–IKZF1^18^ are rather unremarkable and thus cannot account for the increase in stability of the ternary complex. Taken together, the aforementioned observations are slowly uncovering the mechanistic intricacies of CRBN-based targeted protein degradation, and all of them highlight the importance that non-additive mechanisms play in these interactions.^12^ There is currently great interest in discovering new molecules able to engage CRBN to selectively degrade pathology-related proteins.^26−33^ It would also be useful to anticipate the range of neo-substrates that could potentially be engaged with any given molecule, both to look for new applications and to prevent undesired side effects.^34−38^ Understanding the physicochemical factors that govern the direct CRBN engagement of its neo-substrates is therefore essential to streamline the development of novel CRBN-based degraders and to make it less reliant on trial and error. In this work, we use biomolecular simulations to evidence that the stabilization effect exerted by lenalidomide in the complex between CRBN and CK1α relies on its ability to increase the structural stability of three key H-bonds at the CRBN–CK1α interface. Using data for four different mutants of CK1α, we demonstrate that the robustness of these three H-bonds directly correlates with the stability of the ternary CRBN–lenalidomide–CK1α complex, even when mutations do not directly disturb the ability of either protein to establish these interactions. The underlying mechanism is proposed to depend on the capacity of lenalidomide to provide hydrophobic shielding to pre-existing protein–protein hydrogen bonds, thus increasing the structural, kinetic and thermodynamic stability of the complex. Beyond its relevance to the design of novel CRBN-based degraders, we anticipate that this may be a general mechanism that can be exploited for the future rational development of MGs.

## Results

### The Presence of Lenalidomide Results in Stronger H-Bond Interactions at the CRBN–CK1α Interface

**Figure 1:**
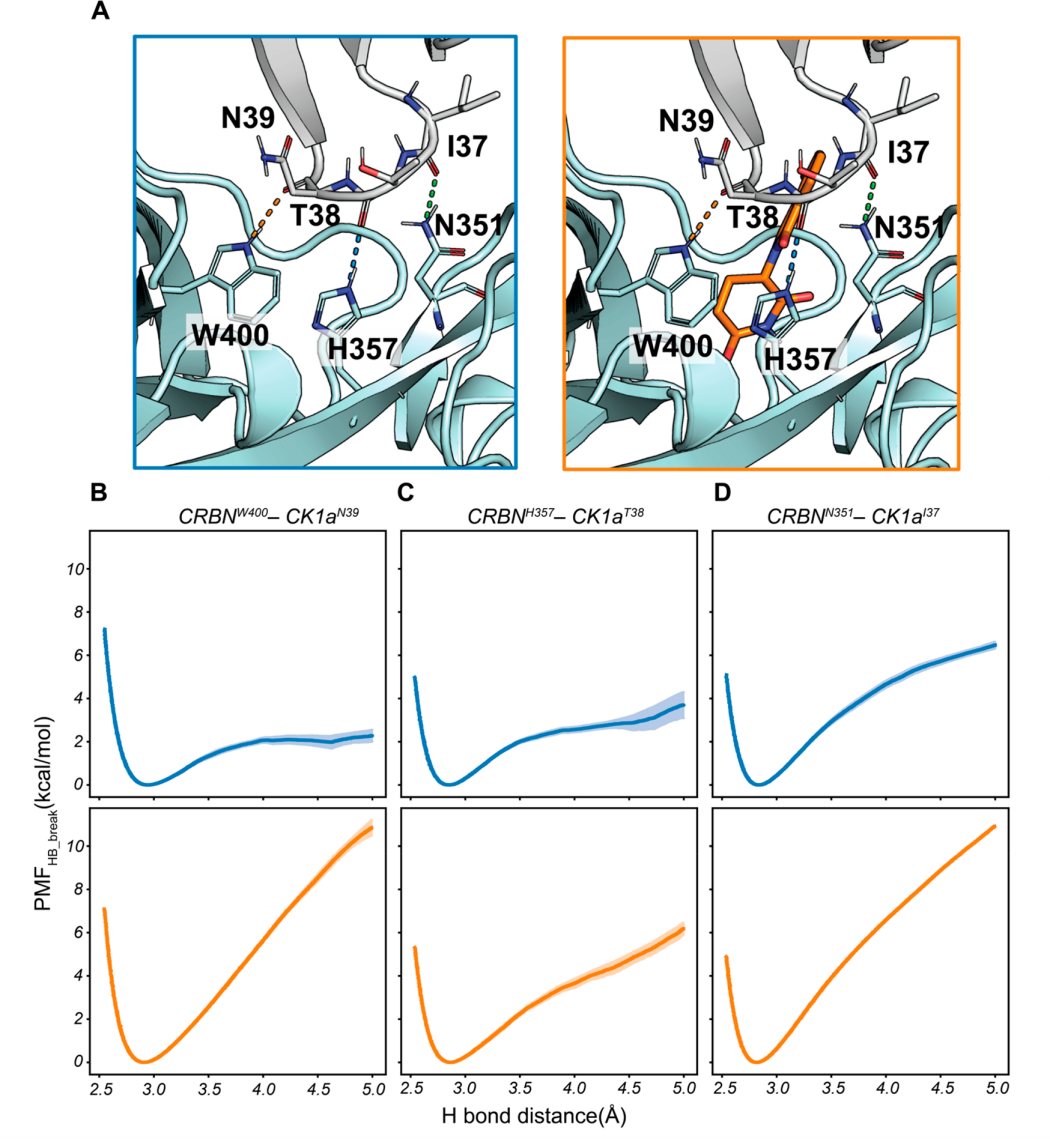
**H-bond dissociation energy profiles in the presence and absence of lenalidomide at the CRBN–CK1α interface.A.** Detailed view of the CK1α–CRBN dimeric interface (left) and its ternary complex with lenalidomide (right). **B.** Energy profile of the CRBN^W400^–CK1α^N39^ H-bond in the absence (top) and presence (bottom) of lenalidomide. **C.** Energy profile of the CRBN^H357^–CK1α^T38^ H-bond in the absence (top) and presence (bottom) of lenalidomide. **D.** Energy profile of the CRBN^N351^–CK1α^I37^ H-bond in the absence (top) and presence (bottom) of lenalidomide. Starting computational models were built from the crystallographic structure with PDB ID: 5FQD.

Examination of the protein–protein interface of the CRBN–lenalidomide–CK1α complex reveals that there are three protein–protein hydrogen bonds between the 36–42 β-hairpin loop of CK1α and the C-terminal domain of CRBN (Figure S1). Namely, the side chains of CRBN residues N351, H357 and W400 engage the backbone carbonyl oxygens of the CK1α residues I37, T38 and N39, respectively. These interactions, hereafter referred to as CRBN^N351^–CK1α^I37^, CRBN^H357^–CK1α^T38^ and CRBN^W400^–CK1α^N39^, respectively, have been demonstrated to be key for the recruitment of CK1α by CRBN.^19^ However, there is no evident factor precluding the formation of these interactions in the absence of lenalidomide, which is in line with the hypothesis of stabilization of pre-existing protein–protein complexes put forward by Cao and co-workers.^25^ Previous works have shown that most stable receptor–ligand complexes display at least one robust and hard-to-break intermolecular H-bond,^39−41^ and the importance of these interactions has also been highlighted as a main player in protein structural stability.^42,43^ Therefore, we investigated the energetic cost of independently breaking each of the H-bonds identified at the CRBN–CK1α interface (Figure 1) combining steered molecular dynamics and the Jarzynski’s equality.^44,45^ In brief, as summarized in Figure S2, after careful equilibration, each H-bond is forced to undergo separation from 2.5 to 5.0 Å, measuring the work needed to carry out the process. For each H-bond, we repeat the steered molecular dynamics 100 times, and the work profiles are Boltzmann-averaged (Jarzynski equality) to obtain an accurate potential of mean force (PMF). Note that the PMF reflects the influence of the whole system (proteins, ligand and solvent) on the interaction that is being assessed.

Although convergence of sampling is usually a concern when applying the Jarzynski relationship, the reduced number of degrees of freedom that the system can access during sampling of the short distance required to break a H-bond (2.5 Å) allows us to calculate the PMF of H-bond breakage (PMF_HB_break_) with sufficient accuracy as to distinguish strong from weak H-bond interactions, similarly to what we and others have previously reported in the literature for other systems.^40,46,47^ By comparing the values of PMF_HB_break_, we established that, in the absence of lenalidomide, the stronger H-bond is CRBN^N351^–CK1α^I37^ (PMF_HB_break_ = 6.4 ± 0.1 kcal/mol), followed by the CRBN^H357^–CK1α^T38^ interaction (PMF_HB_break_ = 3.7 ± 0.6 kcal/mol) and the CRBN^W400^–CK1α^N39^ interaction (PMF_HB_break_ = 2.3 ± 0.3 kcal/mol). The presence of lenalidomide at the interface causes a large increase in the energy necessary to break the three H-bonds, with estimated PMF_HB_break_ = 10.9 ± 0.1, 6.3 ± 0.3 and 11.0 ± 0.4 kcal/mol for the CRBN^N351^–CK1α^I37^, CRBN^H357^–CK1α^T38^ and CRBN^W400^–CK1α^N39^ interactions, respectively (Table 1). The higher energy required to break the interactions results in an increased stiffness of the three H-bonds. This effect is reflected in the narrower distribution of interaction lengths at the interface displayed by the ternary complex with respect to the CRBN–CK1α system during three independent equilibrium MD trajectories of 100 ns (Figure S3). We examined the 3D structure of the ternary complex to obtain clues about the stabilization of the investigated H-bonds. The only direct H-bond between lenalidomide and CRBN (the carbonyl group of the oxoisoindol moiety with the side chain of N351) is insufficiently connected to the protein–protein H-bonds to suggest that it can cause a concerted change in the interaction network. Instead, the increased stability may be explained by the change of local environment around the H-bonds. Indeed, it has been previously shown that incoming water molecules catalyze the rupture of solvent-exposed H-bonds by decreasing the energetic barrier required to bring apart donor and acceptor.^39,48^ Based on these observations, we hypothesized that the main role of lenalidomide will be to create a hydrophobic environment around the protein–protein interface that effectively shields the H-bonds from incoming water molecules.

**Table 1:** Summary of the Absolute and Relative PMF_HB_break_ Values (in kcal mol^–1^) for the CRBN–CK1α Systems Considered in This Work^a^

**H-bond**		^**wt**^**CK1α,**				
**(CRBN–CK1α)**	^**wt**^**CK1α**	**No LEN**	^**I35G**^**CK1α**	^**I37E**^**CK1α**	^**N39G**^**CK1α**	^**G40N**^**CK1α**
W400–N39	10.9 ± 0.3	2.3 ± 0.3	10.3 ± 0.3	10.7 ± 0.3	6.3 ± 0.4	–0.4 ± 1.2
		(−8.6 ± 0.6)	(−0.6 ± 0.6)	(−0.2 ± 0.6)	(−4.6 ± 0.7)	(−11.3 ± 1.5)
H357–T38	6.2 ± 0.3	3.7 ± 0.6	5.8 ± 0.2	5.2 ± 0.2	4.5 ± 0.1	6.2 ± 0.3
		(−2.5 ± 0.9)	(−0.4 ± 0.5)	(−1.0 ± 0.5)	(−1.7 ± 0.4)	(0.0 ± 0.6)
N351–I37	10.9 ± 0.1	6.5 ± 0.2	8.4 ± 0.2	6.8 ± 0.3	6.2 ± 0.6	5.8 ± 1.0
		(−4.4 ± 0.3)	(−2.5 ± 0.3)	(−4.1 ± 0.4)	(−4.7 ± 0.7)	(−5.1 ± 1.1)

**∑PMF**_**HB_break**_	28.0 ± 0.7	12.4 ± 1.1	24.5 ± 0.7	22.7 ± 0.8	16.9 ± 1.1	12.4 ± 2.4
		(−15.6 ± 1.8)	(−3.5 ± 1.4)	(−5.3 ± 1.5)	(−11.1 ± 1.8)	(−15.6 ± 3.1)

a Relative values (in parentheses) are shown with respect to the CRBN–LEN–CK1α complex. Error estimates were obtained by bootstrapping 10 times the W profiles used to estimate the PMF.

### Reinforced H-Bonds Display Increased Hydrophobic Shielding at the CRBN–CK1α Interface

To probe our hypothesis that lenalidomide stabilizes the CRBN–CK1α complex mainly by hydrophobic shielding^49^ effects, we studied the changes in the local environment of the three key H-bonds at the interface upon binding of the MG. The radial distribution function (RDF) provides the average number of water molecules found around a certain atom with respect to what would be expected on the bulk solvent during the course of a molecular dynamics simulation.

**Figure 2:**
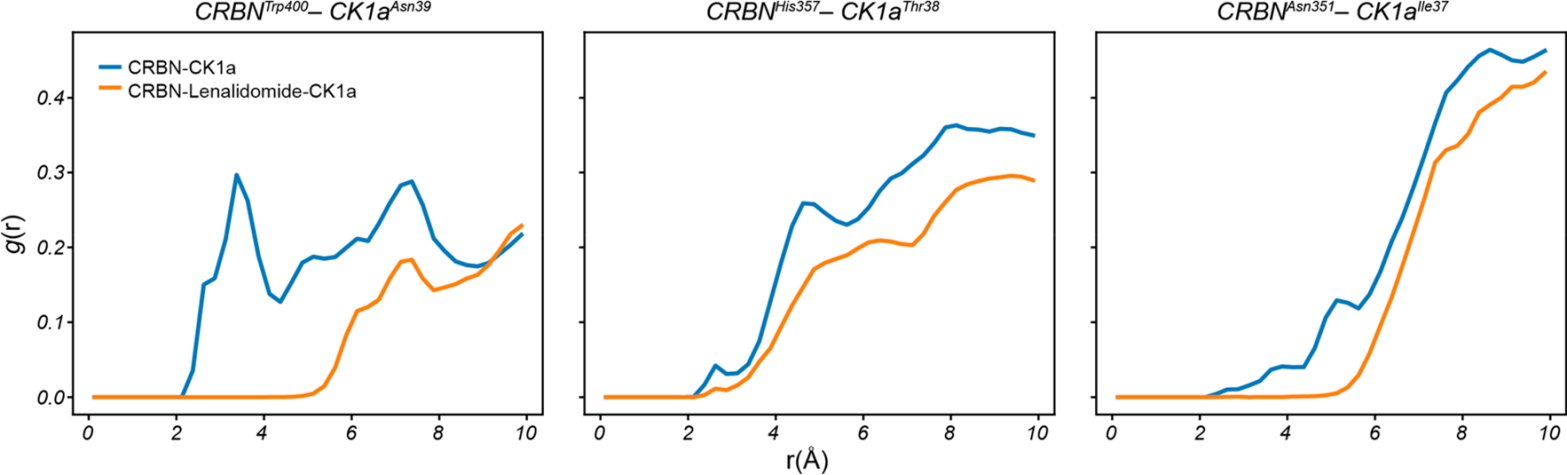
**Radial distribution function (RDF) of water molecules around the backbone carbonyl oxygen of CK1α involved in the key CRBN–CK1α H-bonds.** The blue line represents values for the two-body complex CRBN–CK1α, and the orange line represents values for the three-body complex CRBN–lenalidomide–CK1α.

Therefore, it can be used as a proxy to estimate the solvent exposure of certain atoms or residues. We determined the RDF of the backbone carbonyl groups of CK1α in molecular dynamics of both the CRBN–CK1α and CRBN–lenalidomide–CK1α complexes (Figure 2), in which the H-bond distances for the three key interactions were kept between 2.5 and 3.5 Å using flat bottom restraints (100 simulations of 10 ns for each of the H-bonds, total aggregated time of 3 μs; see the Methods section for further details). As expected for atoms at the interface of the protein–protein complex, their water exposure is relatively low. However, there was a noticeable reduction in the RDF around the backbone carbonyl of N39 (from 0.3 in the binary complex to 0 in the ternary complex). Furthermore, the RDFs around the CRBN^N351^–CK1α^I37^ and the CRBN^W400^–CK1α^N39^ H-bonds for radii below 5 Å drop to 0 in the presence of lenalidomide. On the other hand, the reduction in the RDF for the CRBN^H357^–CK1α^T38^ H-bond—the interaction that is least reinforced by the presence of lenalidomide—is relatively minor.

### H-Bond Robustness Correlates with the Measured Stability of CRBN–Lenalidomide–^MUT^CK1α Complexes

**Figure 3:**
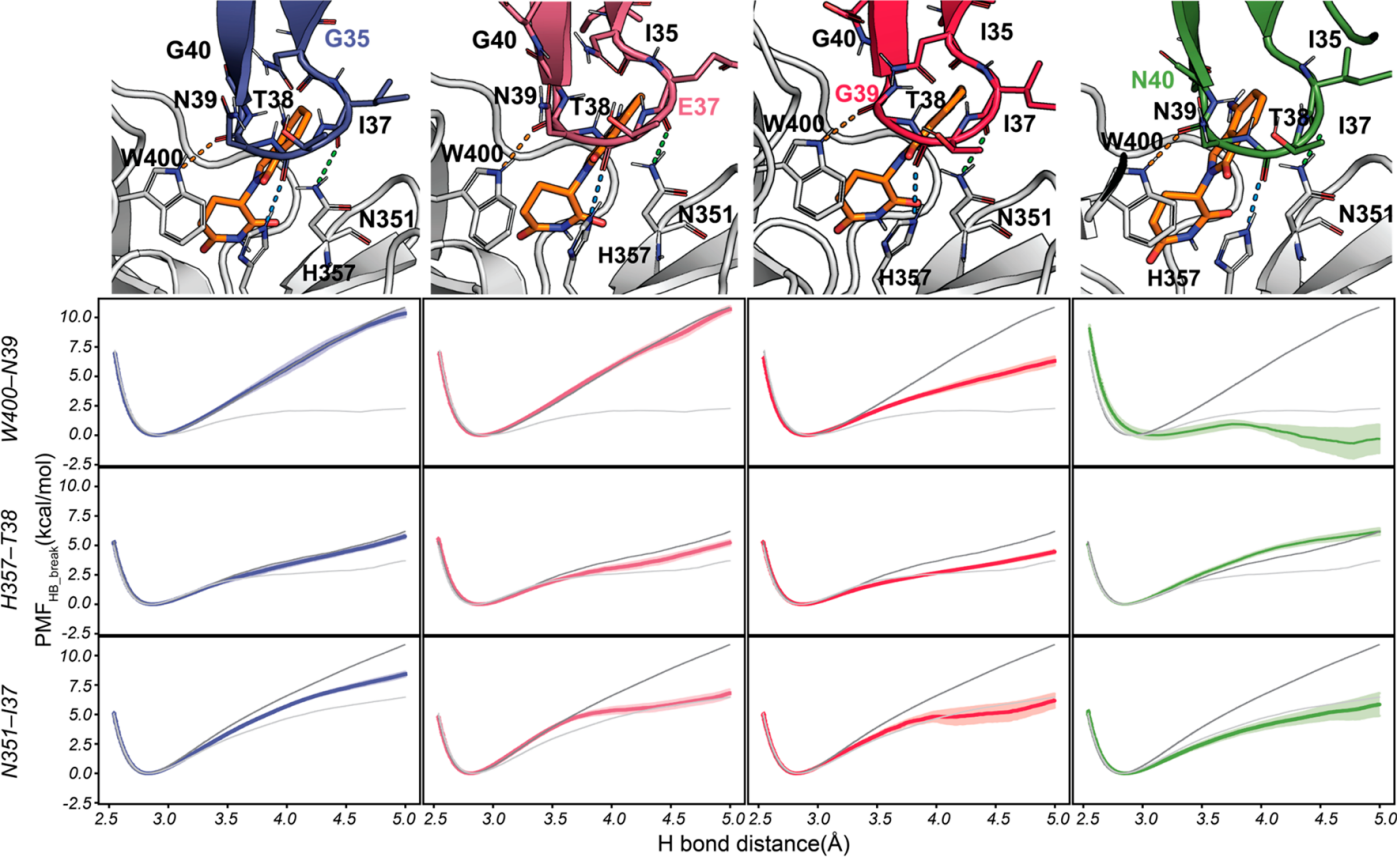
**H-bond dissociation energy profiles in the presence and absence of lenalidomide at the CRBN–CK1α interface.** Detailed view of the CRBN–lenalidomide–CK1α interface for ^I35G^CK1α, ^I37E^CK1α, ^N39G^CK1α and ^G40N^CK1α and associated PMF_HB_break_ profiles for CRBN^W400^–CK1α^N39^ (top), CRBN^H357^–CK1α^T38^ (middle) and CRBN^N351^–CK1α^I37^ (bottom). PMF_HB_break_ profiles for CRBN–lenalidomide–^wt^CK1α (dark gray) and the CRBN–^wt^CK1α (light gray) are included for reference. Starting computational models were built from the crystallographic structure with PDB ID: 5FQD.

Petzold et al. reported that the ternary complexes between CRBN–lenalidomide and CK1α mutants ^I35G^CK1α, ^I37E^CK1α, ^N39G^CK1α and ^G40N^CK1α displayed decreasing stability.^19^ On the one hand, residue 40 in CK1α is analogous to residue N62 in CK2α, which is not susceptible to IMiD-induced CRBN-dependent degradation. On the other hand, residues 35–39 are the CK1α residues located at the interface with lenalidomide and CRBN, but, as mentioned, polar interactions with CRBN are established through the carbonyl oxygens of the protein backbone, while the side chains seem to only establish non-specific van der Waals contacts; hence, the stability of the ternary complex should be relatively insensitive to the nature of the side chain. Furthermore, the side chain of the glutamate residue in the ^I37E^CK1α could be expected to establish additional polar contacts with CRBN residues H353 and Y355. Consequently, the decrease in stability of the ternary complex for the mutants of CK1α was not easily anticipated from the structural data. We therefore investigated whether the robustness of the H-bonds at the interface on these ternary complexes was diminished with respect to that of the CRBN–lenalidomide–CK1α complex (Figure 3 and Table 1).

Analysis of the energetic profiles of H-bond breakage showed that ^N39G^CK1α and ^G40N^CK1α displayed the greatest alterations, both with ΔPMF_HB_break_ in excess of 4 kcal/mol not only for the proximal CRBN^W400^–CK1α^N39^ interaction but also for the distal CRBN^N351^–CK1α^I37^ H-bond. In the case of the CRBN^H357^–CK1α^T38^ interaction, there was a lesser reduction in ^N39G^CK1α (ΔPMF_HB_break_ = 1.7 kcal/mol), while in the case of ^G40N^CK1α, the latter interaction was not affected. In fact, besides ^N39G^CK1α, the effect of mutations on the CRBN^H357^–CK1α^T38^ interaction was within the estimated uncertainty margins (ΔPMF_HB_break_ = 0.0 ± 0.6, −0.4 ± 0.5 and 1.0 ± 0.5 kcal/mol for the ^G40N^CK1α, ^I35G^CK1α and ^I37E^CK1α mutants, respectively). For the ^I35G^CK1α and ^I37E^CK1α mutants, only the CRBN^N351^–CK1α^I37^ was significantly weakened with respect to the wild-type (*wt*) complex, displaying ΔPMF_HB_break_ = 2.5 ± 0.3 kcal/mol and 4.1 ± 0.4 kcal/mol, respectively. Therefore, all mutants displayed at least equally but often more robust H-bonds than the complex between CRBN and CK1α without lenalidomide, but the breaking energy profile with respect to the *wt* ternary complex was weakened for at least one of the H-bonds in all the cases.

**Figure 4:**
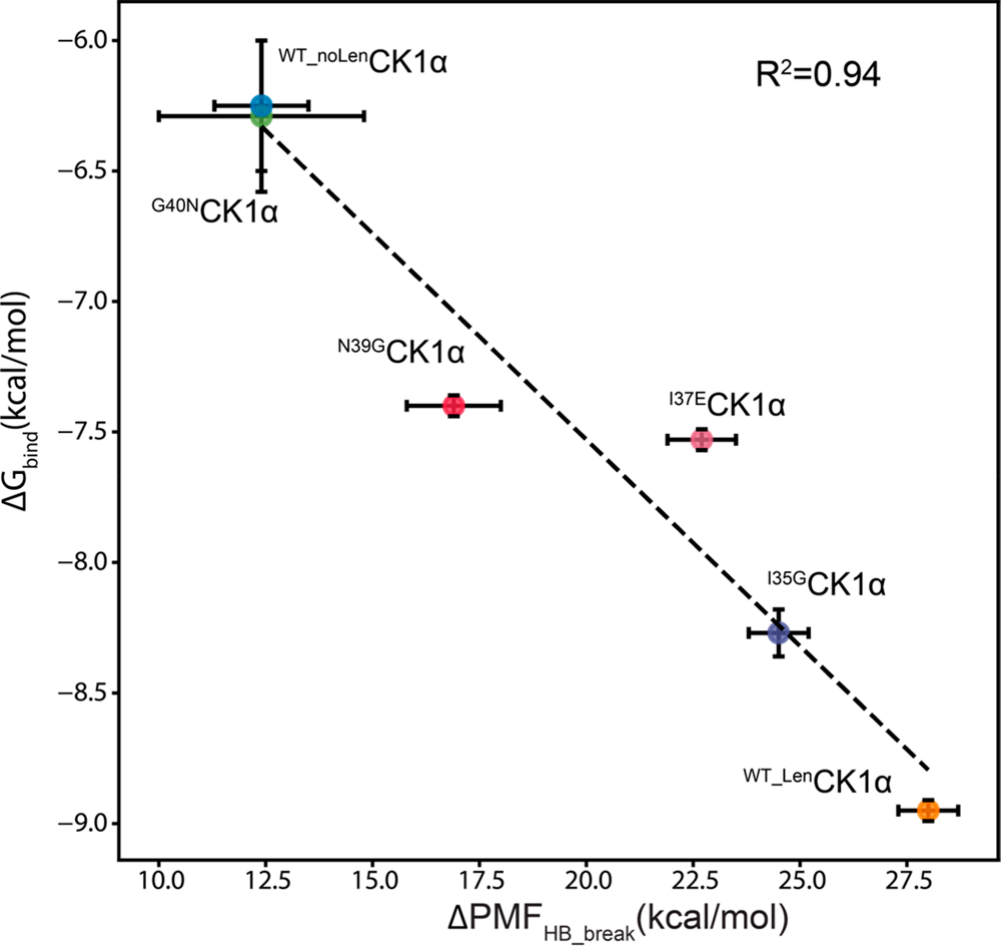
**Correlation plot between the sum of the PMF**_**HB_break**_**values for the three H-bonds for different variants of CK1α with respect to Δ*G***_**bin**_**calculated from *K***_**D**_**estimations.** The PMF_HB_break_ was taken at the end point of the PMF profile, and error bars were obtained by bootstrapping of the W profiles. PMF_HB_break_ values are reported in Table 1. Δ*G*_bin_ was obtained by transforming the *K*_D_ values fitted using data from ref (19), and error bars were obtained by error propagation. The reproduced [CRBN]–520/490 nm TR-FRET ratio plot is provided in Figure S10, and experimental values are provided in Tables S1 and S2.

While there is no experimental value that can be linked directly with the calculated ΔPMF_HB_break_ for the breaking of singular H-bonds, we hypothesized that the observed variation in the energy required for breaking the three interactions at the CRBN–CK1α interface may inform about the stability of the resulting ternary complex with lenalidomide. To probe this possibility, we first established that there was no co-dependence between the breakages of the three hydrogen bonds (Figure S4); therefore, the energy required to break all three bonds could be approximated by adding the individual PMF_HB_break_ values. We found that the sum of PMF_HB_break_ for the three key H-bonds in each of the complexes between CRBN and CK1α was correlated with its estimated binding affinity (*R*^2^ = 0.94, Figure 4).

### Weakening of the Three H-Bond Interactions Stems from Better Accessibility of Water Molecules to the Protein–Protein Interface

Having established the correlation between the strength of the hydrogen bonds at the CRBN–lenalidomide–CK1α interface and the stability of the ternary complex, we next investigated the molecular determinants that could account for the reduced strength of the hydrogen bonds displayed by the four single-point CK1α mutants. We determined the RDF of water molecules around the H-bonds and compared them with the RDF profiles obtained for the binary and ternary complexes of CK1α (Figure S5). All the mutants displayed RDF profiles closer to the ternary complex than to the binary complex. Nevertheless, the profile obtained for the ternary complex involving the ^N39G^CK1α mutant was very different from the one obtained for the *wt* CK1α, with increased RDF values with respect to the latter in the areas of the first and second solvation shells for both the CRBN^H357^–CK1α^T38^ and the CRBN^W400^–CK1α^N39^ H-bonds, while the remaining H-bond (the farthest from the mutation point) only displayed differences beyond 6 Å. Similar patterns were observed in the profiles obtained for the ^I35G^CK1α and ^I37E^CK1α mutants, where the closest H-bond was the most affected by the change, although in these cases the differences were only observed in the second solvation shell region. In contrast with the stark decrease in PMF_HB_break_, the profiles for the remaining mutant ^G40N^CK1α were indistinguishable from the profiles of the ternary complex with the *wt* CK1α. Intrigued by this apparent discrepancy, we visualized the trajectories and identified that, regardless of the system involved, low breaking profiles corresponded with those in which at least one water molecule entered the protein–protein interface from the bulk and established an H-bond with the carbonyl atom previously involved in the protein–protein interaction, while high work profiles corresponded with H-bond breakages in which water molecules did not access the protein–protein interface or did not establish an H-bond (Supplementary Movie S1). While loss of hydrophobic shielding of the protein–protein hydrogen bonds is ultimately responsible for the loss of stability, the subjacent causes may be diverse and non-obvious. For the mutations involving introduction of a glycine residue (I35G and N39G), a change in the intrinsic conformational preferences of the loop may partially explain our results (Figure S6). For ^I37E^CK1α, the charged side chain perturbs the local water distribution, particularly in the second shell of solvation (Figure S5), and it is conceivable that it facilitates hydration of the N351–I37 hydrogen bond once it starts to dissociate. For ^G40N^CK1α, we hypothesized that the higher rate of access of water molecules to the protein–protein interface in the case of the ^G40N^CK1α may be related to a worse hydrophobic packing of lenalidomide’s core against the bulkier and more flexible asparagine side chain than against the glycine residue in position 40. We therefore measured the average distance between lenalidomide’s center of mass and the α carbon of residue 40 of CK1α in all the mutants and in the *wt* (Figure S7). The average distance was estimated to be ca. 4.8 Å for all the systems but ^G40N^CK1α, in which the average distance was closer to 5.8 Å.

**Figure 5:**
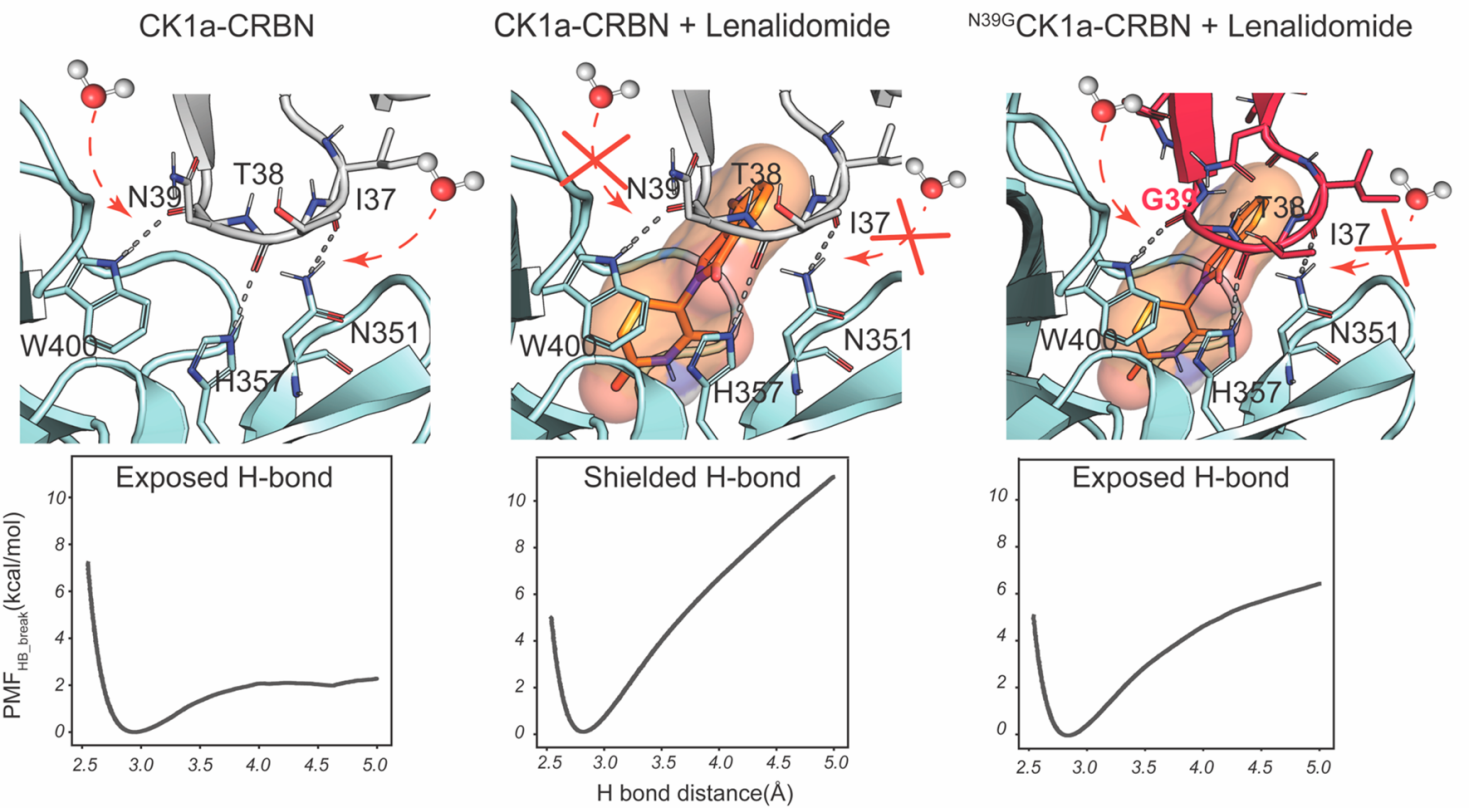
**Proposed mechanism underlying the stabilization of the CRBN–CK1α complex by lenalidomide and the effect of mutations in the CK1α sequence.** Lenalidomide hinders water accessibility to the CRBN–CK1α interface, increasing the strength of H-bonds. Mutations that alter water accessibility to the interface diminish the stability of the ternary complex. Starting computational models were built from the crystallographic structure with PDB ID: 5FQD.

Considering the results, we propose that lenalidomide (and by extension other IMiDs) enables the degradation of CK1α and other CRBN neo-substrates by strengthening the pre-existing H-bonds at the interface, which results in a complex stable enough as to be tagged by ubiquitination (Figure 5). The reinforcement of the H-bonds seems to be related to the ability of IMiDs to hinder access of water molecules to the protein–protein interface, and hence, their effectiveness is very susceptible to single-point mutations that increase the flow of water into the interface, by means of either local or long-range effects (Figure 5). The parameters controlling solvent accessibility can vary from one system to the next, and they may affect the bound (equilibrium) state or may only become apparent during the dissociation process (out of equilibrium), but in all cases, the free energy of hydrogen bond rupture can be readily calculated using SMD simulations.

## Discussion

This work demonstrates that the presence of lenalidomide at the CRBN–CK1α interface results in a significant increase in the free energy required to break three key H-bond interactions at the protein–protein interface (Figure 1). It also highlights the sensitivity of this effect to point mutations, even when they do not directly hinder the formation of the H-bonds (Figure 3 and Table 1). Interestingly, we detect an important correlation (squared Pearson *R* value of 0.94) between the cumulative strength of the three hydrogen bonds and the energy of binding derived from the observed *K*_D_. In principle, there is no reason why the binding energies derived from *K*_D_ measurements (an equilibrium property) and the breaking energies of H-bonds (which as computed are an out of equilibrium property) should be correlated. However, we propose that the correlation is not spurious and instead reflects two key mechanistic aspects of the interaction between CRBN and CK1α. First is that the CK1α point mutations studied are not likely to affect the *k*_on_ of the complexes, which makes the observed decreases of affinity almost exclusively dependent on changes of the *k*_off_. Second, and more crucially, the outstanding correlation between the free energy of H-bonds rupture and the observed affinity indicates that the dissociation of these complexes follows a rather simple two-state mechanism, where breaking the H-bonds at the interface is the rate-limiting step. Under these circumstances, the PMF_HB_break_ is the major contributor to changes in the *k*_off_ and can inform about the equilibrium constant. This observation, together with the dramatic effect that lenalidomide has on the stability of the interaction between CRBN and CK1α (>100 fold decrease in apparent *K*_D_), underscores the potential that rationally designed MGs could hold for the modulation of protein–protein interactions in biomedical and biotechnological settings.

Regarding the underlying mechanism, deconvoluting all the different factors that result in increased or decreased PMF profiles is not straightforward because they encapsulate effects derived from both bonded (such as backbone flexibility or side-chain rotamer preferences) and non-bonded (such as water accessibility to the interacting groups) contributions. In practice, this means that the resulting PMF profiles capture subtle differences in protein behavior that are difficult to anticipate from structural analysis. For example, our results indicate that mutations to glycine may result in a more flexible loop in CKα that may partially account for the decreased stability of the ternary complex with respect to the *wt*. However, backbone flexibility cannot explain the decrease in the stability of the ternary complex involving ^G40N^CK1α and ^I37E^CK1α (Figure S5). In that sense, we have shown that, when bound to the CRBN–CK1α interface, lenalidomide severely hinders water accessibility to the key protein–protein hydrogen bonds, as demonstrated by the stark decrease in the RDF value (Figure 2). This hydrophobic shielding effect seems to be a main driver in the stabilization effect triggered by lenalidomide, and thus it could be considered to play a major role in the non-additive effects observed for this compound. It has been previously reported that relatively minor alterations of the H-bond environment can significantly alter H-bond lifetimes.^39,48,50^ This effect is also entirely consistent with the stabilization of pre-existing interactions put forward by Cao and co-workers,^25^ and it is expected that a similar mechanism underlies the degradation of other CRBN neo-substrates such as Ikaros and Aiolos and is shared by other IMiDs such as pomalidomide (Figure 5). Beyond CRBN-related systems, by analyzing the crystallographic structures available in the PDB, we hypothesize that a similar effect underlies the recently described cannabidiol-dependent stabilization of a dual-nanobody sensor^25^ (PDB ID: 7TE8) and the long-standing puzzle of the fucsicoccin-dependent stabilization of interactions involving 14-3-3 proteins (PDB ID: 3P1S)^51^ (Figure S8). Interestingly, evaluating water accessibility to the protein interface is not enough to anticipate H-bond strength. While the changes triggered by the ^I35G^CK1α, ^I37E^CK1α and ^N39G^CK1α mutations can be rationalized on the basis of local changes to the environment of the H-bond, the behavior observed for ^G40N^CK1α is rather unexpected, as an increase in the size and hydrophobicity of the side chain results in better access of water molecules to the protein–protein interface during the H-bond rupture process that is not anticipated by RDF profiles of the complexes in equilibrium. The effect of the G40N mutation is also in contrast with the effect of the N39G mutation, given that the reverse mutation yields a similar outcome. Therefore, our results stress that, though often neglected, changes in the protein–protein interactions caused by the presence of MGs are as important as the direct interactions between the MGs and the proteins. We propose that our results complement the most recent discoveries in the function and structure–activity relationships for CRBN,^22−24^ describing an atomistic rationale that anticipates the susceptibility of a given neo-substrate to form a stable ternary complex with CRBN and a putative MG. We postulate that instead of solely focusing on maximizing affinity, computer-aided drug design strategies for MGs should also aim at maximizing protein–protein interactions by hydrophobic shielding of polar interactions. Analogous strategies should also be investigated for other types of interactions. In this work we demonstrate that an easy-to-implement SMD-based protocol is enough to predict stabilization of H-bonds which, in this particular system, offer an excellent predictor of the thermodynamic stability of the ternary complex. Nevertheless, our work focuses on a particular stage of the CRBN-dependent degradation mechanism and, therefore, is an additional piece of a complex puzzle that has to account for other effects before and after the ternary complex formation, such as allosteric effects or degradation susceptibility of the neo-substrate. It also remains to be investigated if these results will transfer to other MGs systems, but the incorporation of the described mechanistic insights into drug design workflows may assist much-needed rational approaches to the design of future MGs.

## Methods

### Molecular Simulations Setup

Lenalidomide was built using the Molecular Operating Environment software package.^52^ Models for the CRBN and CK1α were built starting from the crystallographic structure PDB ID: 5FQD,^53^ downloaded from the Protein Data Bank.^54−56^ Standard protein preparation protocols were followed, including the removal of duplicated proteins, crystallization buffer compounds and salts. Additionally, the DNA damage-binding protein 1 was removed in all systems, and the appropriate capping groups were added to the terminal residues of CRBN. Mutants of CK1α were obtained with the mutagenesis wizard tool in PyMOl.^57,58^ The ff14SB^59^ and gaff2^60^ force fields were used to assign atom types for the protein and the lenalidomide, respectively. Partial charges for lenalidomide were derived using the RESP^61,62^ protocol at the HF/6-31G(d) level of theory, as calculated with Gaussian09. The Zn^2+^ cation bound to CRBN was modeled using the out-of-center dummy model.^63^ Each system was solvated on a truncated octahedral box of TIP3P^64,65^ water molecules, and the appropriate numbers of counterions were added to achieve charge neutrality, accounting for simulation systems of approximately 100 000 atoms. Each system was then minimized in three stages: First, the position of water molecules was minimized, combining 3500 steps of steepest descent and 6500 steps of conjugate gradient, while the position of the proteins and ligand atoms was restrained using a harmonic potential with force constant of 5.0 kcal mol^–1^ Å^–2^. Next, side chains and water molecules were minimized using 4500 steps of steepest descent, followed by 7500 steps of conjugate gradient, while the atoms of lenalidomide and the Zn^2+^ cation were restrained with a harmonic potential using the same force constant. The systems were then heated in the NVT ensemble from 100 to 298 K in three stages of 250 ps (100–150 K, 150–250 K, 250–298 K), while retaining the harmonic restraints to lenalidomide and the Zn^2+^ cation, and subsequently their density was equilibrated to 1 bar for 1 ns in the NPT ensemble. During the equilibration and subsequent production and steered molecular dynamics trajectories, temperature control was achieved using a Langevin thermostat (with a collision frequency of 3 ps^–1^), and a Berendesen barostat was used to control the pressure when simulating in the NPT ensemble. SHAKE^66^ was applied to all atoms involving hydrogen to allow for a time step of 2 fs, and all simulations were performed with the CUDA accelerated version of PMEMD.^67^

### Equilibrium Molecular Dynamics Protocol

The equilibrium MD trajectories for the CRBN–CK1α, CRBN–CK1α–Lenalidomide and the ^MUT^CK1α were run using a common protocol. In brief, three independent trajectories with velocities assigned at 298 K using different seed numbers were started from the equilibrated structures. Each production run consisted of 100 ns (complex simulations) or 200 ns (CK1α systems) run in the NPT ensemble, using a Langevin thermostat and a Montecarlo barostat to control temperature and pressure, respectively. SHAKE^66^ was applied to all atoms involving hydrogen to allow for a time step of 2 fs, and all simulations were performed with the CUDA accelerated version of PMEMD.^67^

### Steered Molecular Dynamics Protocol

The stability of each H-bond in each system was assessed using 100 independent canonical ensemble SMD trajectories conducted in three stages (Figure S2). First, new velocities were assigned to the equilibrated structure using a different random seed number at 298 K and a MD trajectory was performed for 10 ns, using flat-bottom restraints to keep the three protein–protein H-bonds at the interface between 2.5 and 3.5 Å, using a force constant of 60 kcal/mol Å^2^. Second, the final configuration of each trajectory was then used as a starting structure for a short (1 ns) SMD simulation in which the donor and acceptor involved in one of the H-bonds were brought to a distance of 2.5 Å. Third, a 5-ns-long SMD trajectory was started, in which the distance between donor and acceptor was increased at a rate of 0.5 Å/ns, using a spring constant of 500 kcal/mol·Å^2^ to ensure the applicability of the stiff spring approximation.^68^ The PMF_HB_break_ was then computed leveraging the Jarzynki’s equality,^69,70^eq 1.
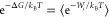
1where the right-hand term corresponds to the ensemble average of exponential work values obtained in non-equilibrium conditions. From the above equation, for every increase of 0.0005 Å in the H-bond distance, the PMF_HB_break_ was obtained using expression 2,
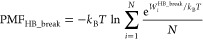
2where *W*_*i*_^HB_break^ refers to the work value of the *i*th independent SMD trajectory and *N* is the number of independent SMD trajectories (*N* = 100 in this work). Error estimations for the PMF_HB_break_ profiles were obtained by bootstrapping 10 times at each distance point the set of *W*^HB_break^ values. Convergence of the PMF_HB_break_ at 5 Å of H-bond (Figure S9) distance was evaluated combining subsampling and bootstrapping.

### Calculation of Water Radial Distribution Function and Ramachandran Plots

The RDF of water molecules around the backbone carbonyl oxygen of the CK1α residues involved in the interaction with CRBN and the Phi/Psi angles of the CK1α loops were calculated using cpptraj.^71,72^ The RDF was calculated from the first step of the SMD protocol (equilibration with restraints to keep all three H-bonds formed), corresponding to a simulation time of 1 μs (100 simulations × 10 ns) per system. The RDF considered the range between 0 and 10 Å from the atom of interest and used a bin spacing value of 0.1 Å.

### Experimental Data Sourcing and Analysis

Time-resolved fluorescence resonance energy transfer (TR-FRET) data points were extracted from Petzold et al.^53^ using WebPlotDigitizer v4.5,^73^ and analysis was performed with the Graphpad Prism 8 software.^74^ Data points were adjusted to a non-linear regression curve, achieving binding saturation. The maximum ratio value obtained for the CRBN–CK1α–lenalidomide ternary complex was used as the constrained maximum signal (*Y*_max_) in all the conditions to determine the *K*_D_.

## Supporting Information Available

Supporting Figures S1–S10 and Tables S1 and S2 (PDF)
Dataset S1 containing output PDB structures, sample input files and unprocessed W profiles used to build the PMF_HB_break_ profiles (ZIP)
Supplementary Movie S1, showing selected SMD trajectories and associated W profiles (MP4)

## Abbreviations Used

CRBN: Cereblon
MG: Molecular Glue
CK1α: Caseine Kinase 1α
IMiD: Inmunomodulatory Drug
PMF: Potential of Mean Force
