# Supplementary material for: Lenalidomide Stabilizes Protein–Protein Complexes by Turning Labile Intermolecular H-Bonds into Robust Interactions
Source: J Med Chem. 2023 Apr 21;66(9):6037–46. doi: 10.1021/acs.jmedchem.2c01692 (PMC10184122; doi:10.1021/acs.jmedchem.2c01692)
Supplement: Supplementary file 1 — jm2c01692_si_001.pdf [file jm2c01692_si_001.pdf]

Supporting Information

Lenalidomide stabilizes protein-protein complexes by turning labile intermolecular H-bonds into robust interactions.

Marina Miñarro-Lleonar,[1,2,3] *Andrea Bertran-Mostazo*[1, 3]*, Jorge Duro,*[1,2] *Xavier Barril,*[1,2,3,4]* *Jordi Juárez-Jiménez,*[1,2]*

[1]Unitat de Fisicoquímica, Departament de Farmàcia i Tecnologia Farmacéutica, i Fisicoquímica. Facultat de Farmacia I Ciències de l'Alimentació. Universitat de Barcelona (UB). Av. Joan XXIII, 27-31, 08028 Barcelona, Spain.

[2]Institut de Química Teòrica i Computacional (IQTC), Facultat de Química i Física, Universitat de Barcelona (UB). C. Martí i Franqués, 1, 08028, Barcelona, Spain.

[3]Institut de Biomedicina, Facultat de Biologia, Universitat de Barcelona (UB), Av. Diagonal, 643, 08028, Barcelona, Spain

[4] Catalan Institution for Research and Advanced Studies (ICREA), Pg. Lluís Companys, 23 08010, Barcelona, Spain.

Corresponding Authors

* Jordi Juárez-Jimenez – Email: jordi.juarez@ub.edu

* Xavier Barril – Email: xbarril@ub.edu

Table of contents

Supplementary Figures

Figure S1 S3

Figure S2 S4

Figure S3 S5

Figure S4 S6

Figure S5 S7

Figure S6 S8

Figure S7 S9

Figure S8 S10

Figure S9 S11

Figure S10 S12

Supplementary Tables

Table S1 S13

Table S2 S14

Other supplementary material S15

Supplementary References S15

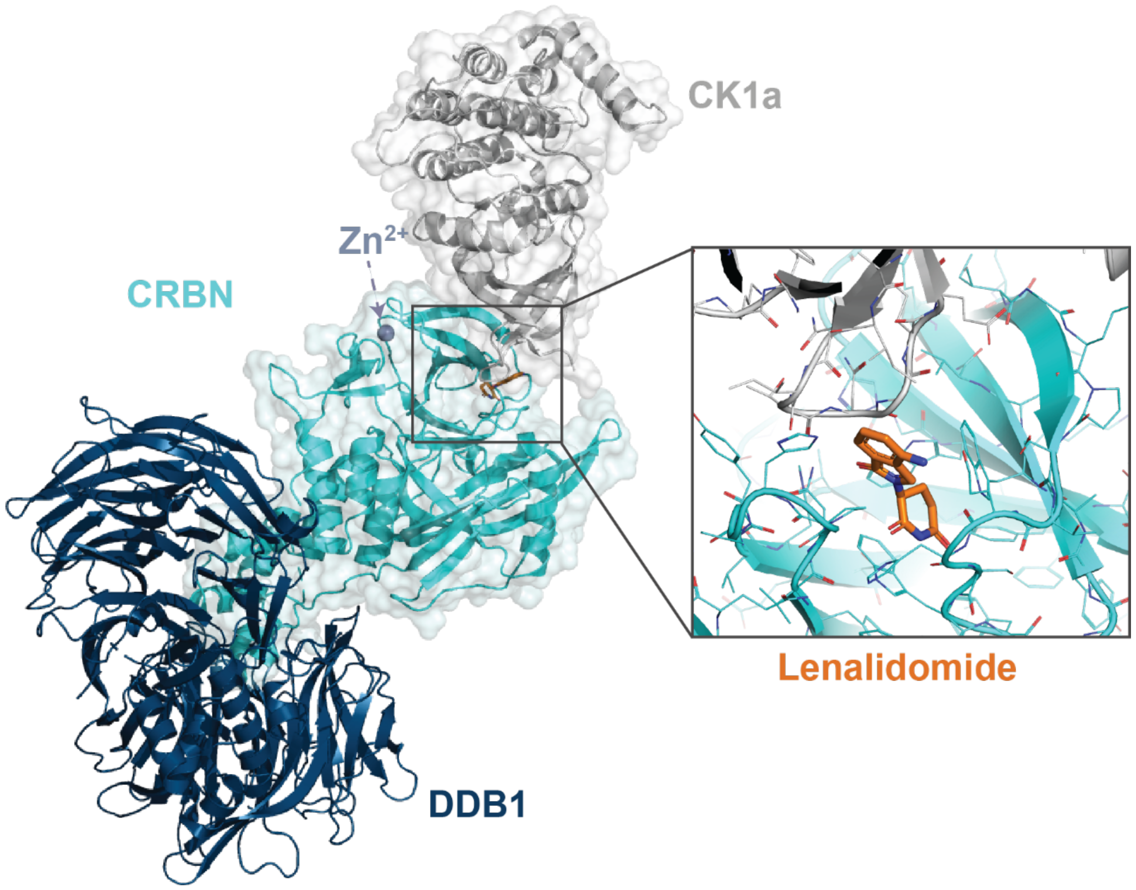

Figure S1: Representation of the complex between the DNA Damage-binding protein 1 (DDB1), the E3 ligase Cereblon (CRBN), the isoform alpha of Casein Kinase 1 (CK1α). Lenalidomide bound to the tryptophan cage of CRBN at the interface with CK1α. Left panel are the trajectories without lenalidomide and right panel are the trajectories with of lenalidomide

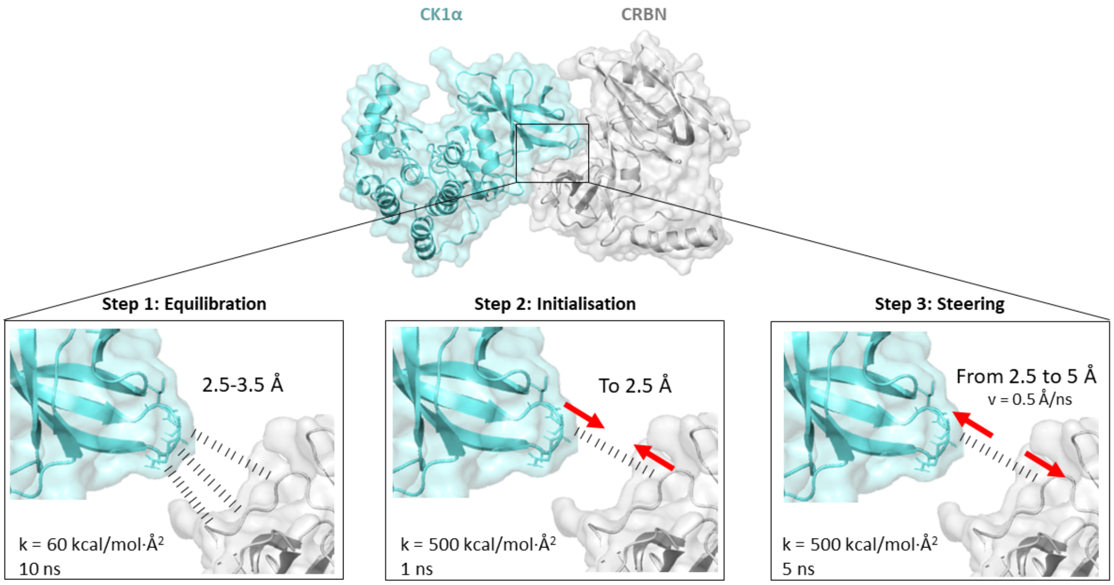

Figure S2: Graphical conceptualisation of the SMD protocol. The breakage of each of the H-bonds stablished between CK1α and CRBN was studied following a three-step protocol. Step 1: equilibration of other degrees of freedom for 10 ns while the three H-bonds at the CK1α–CRBN interface were restrained to a distance between 2.5 and 3.5 Å (restraint force constant: 60 kcal mol^{-1} $Å^{-2}$). Step 2: The H-bond to be broken was brought to a distance of 2.5Å in 1 ns of SMD (restraint force constant: 500 kcal mol^{-1} $Å^{-2}$). Step 3: the H-bond distance was pulled from 2.5Å to 5 Å in 5 ns of SMD (pulling speed: 0.5 Å/ns, restraint force constant: 500 kcal mol^{-1} $Å^{-2}$). This protocol was repeated 100 times for each H-bond in each system.

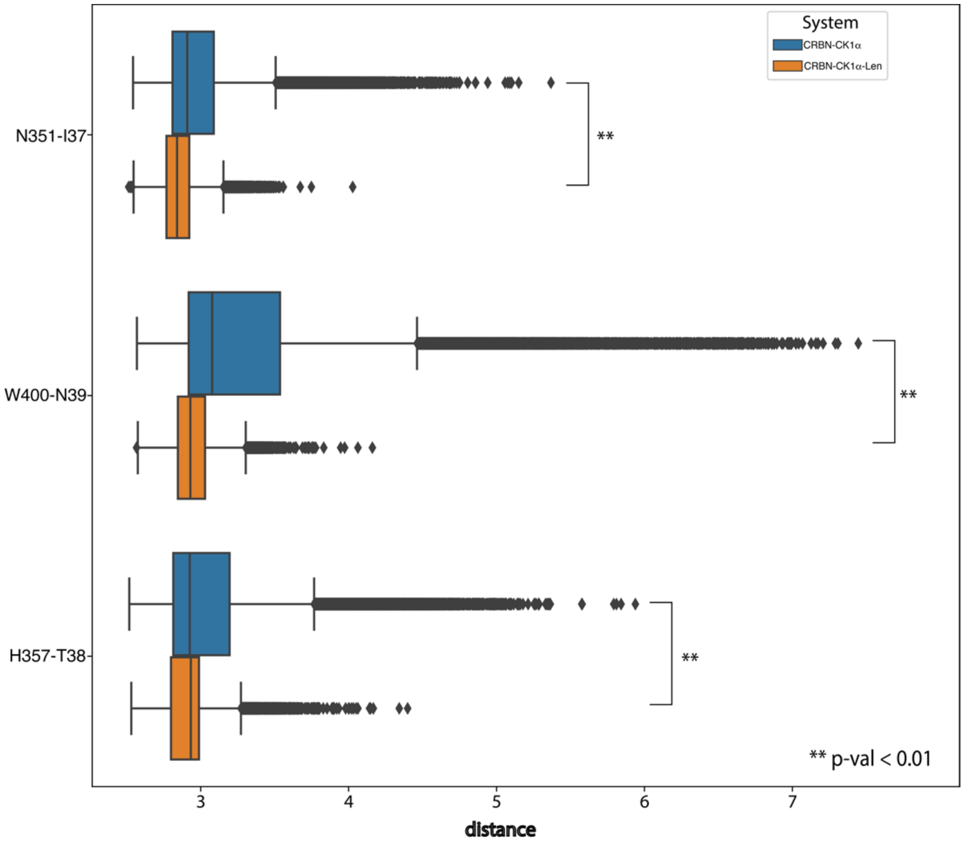

Figure S3: Distribution of distance values of the three H-bonds at the CRBN–CK1α interface. Data was obtained during the course of three independent equilibrium MD trajectories of 100 ns for each system (total aggregated sampling time 300 ns for each system). A Welch t-test shows statistically significant differences in the three H-bonds when comparing the CRBN–CK1α complex and the ternary complex with Lenalidomide.

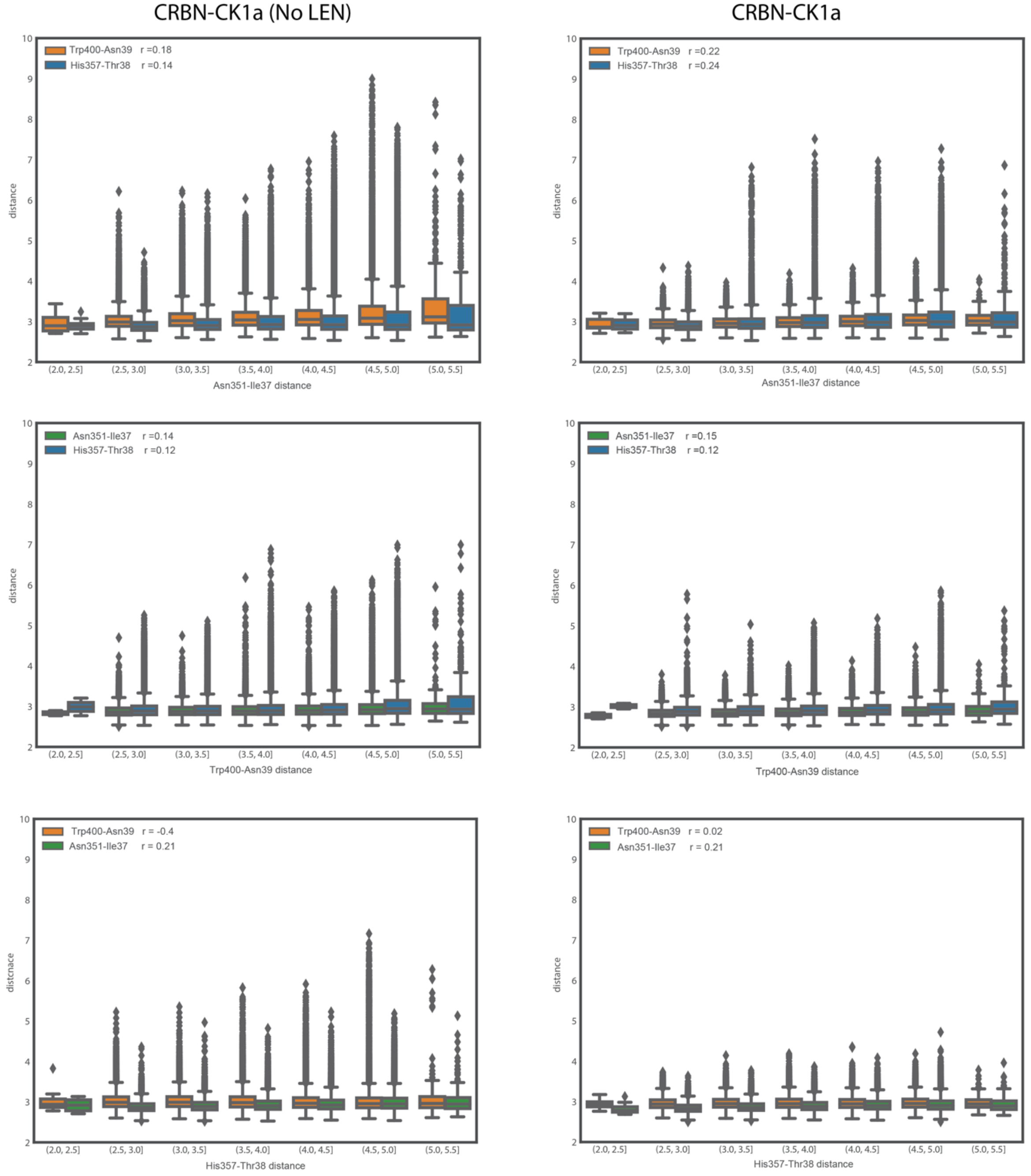

Figure S4: Distribution of distance values of the two H-bonds at the CRBN–CK1α interface when the third is pulled apart in SMD trajectories. Left panel are the trajectories without lenalidomide, and right panel are the trajectories with of lenalidomide. Average Pearson correlation coefficients (obtained from the inverse of the average Fisher's transformed correlation coefficients from individual trajectories) are shown.

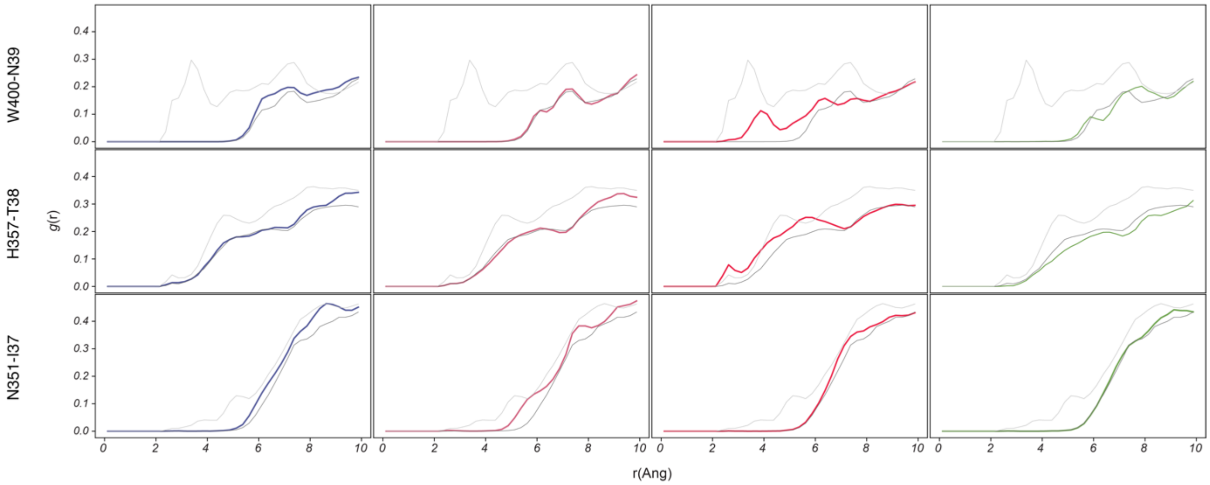

Figure S5: RDF values around the backbone carbonyl oxygen of CK1α residues 37, 38 and 39 for each of the complexes of CRBN with the CK1α mutants considered. ^{I35G}CK1α is depicted in marine, ^{I37E}CK1α is depicted in magenta, ^{N39G}CK1α is depicted in red and ^{G40N}CK1α is depicted in green. The profiles for the complex of ^{wt}CK1α with CRBN in the presence (black) and in the absence (grey) of lenalidomide are also shown for reference

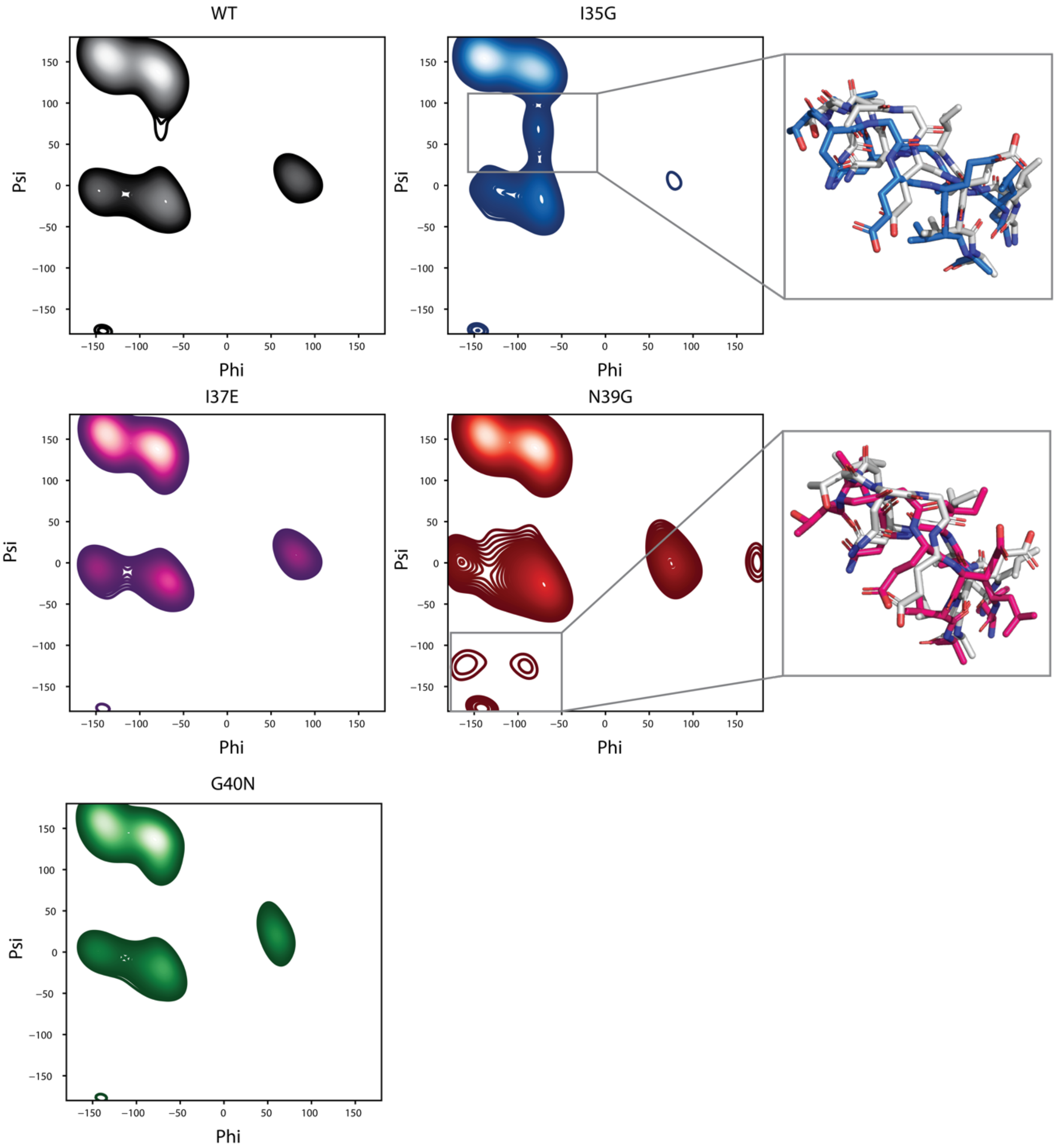

Figure S6: Ramarchandran plots for the 33-43 loop residues in CK1α. Populations were calculated over three equilibrium MD trajectories of 200 ns each, run for ^{wt}CK1α and the four mutants. Details on right compare the loop conformation of the mutant (colored structure) with respect the conformation of ^{wt}CK1α in the crystal structure with PDB ID: 5FQD.

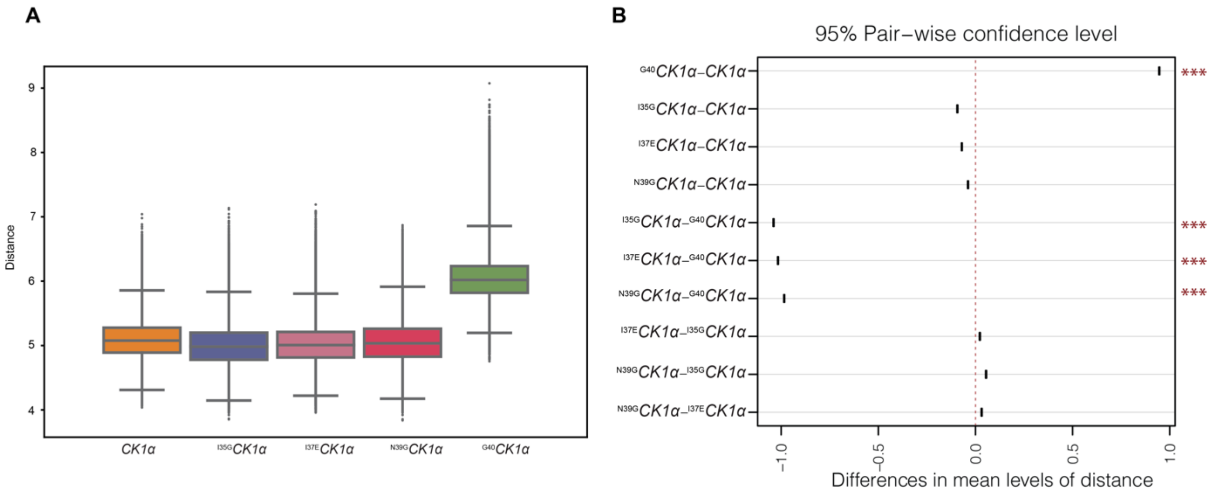

Figure S7: Comparison of the distance distribution between the COM of Lenalidomide and the CA of residue 40 in the different ternary complexes considered in this work. A. Populations were calculated over all the unbiased MD trajectories performed in the first stage of the SMD sampling protocol (10 ns x 100 trajectories, see Methods sections for details). An ANOVA test shown statistically significant differences among the distributions of distance values (for a $P<0.001$). **B.** A Tukey test confirmed that the mean value for the complex involving ^{G40N}CK1α was different to the other populations.

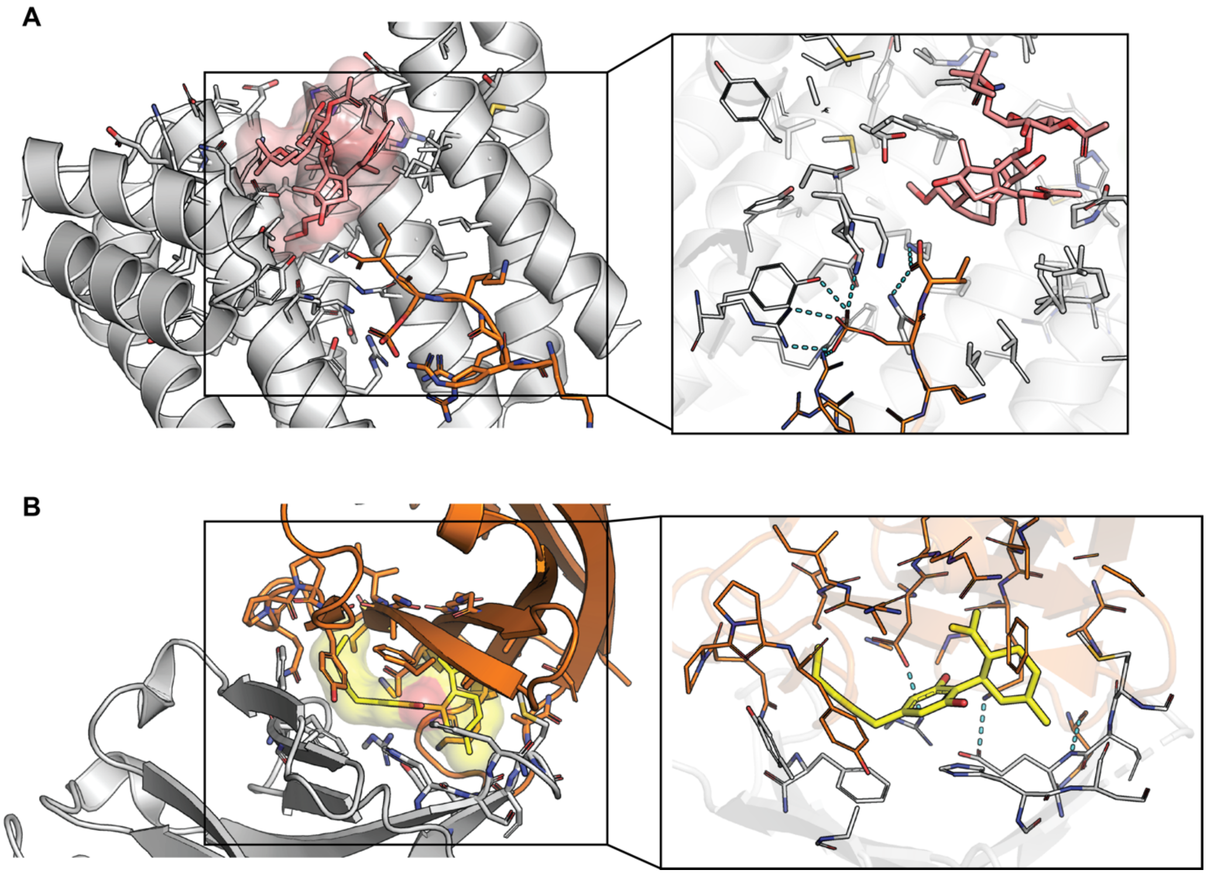

Figure S8: Impaired water accessibility to the protein-protein interface is hypothesized to underlie other MGs dependent ternary complexes such as A. The complex between 14–3–3 and TASK3 stabilised by fusicoccin A (PDB id: 3P1S)[1] and **B.** The complex cannabidiol dependent complex between two engineered nanobodies (PDB id: 7TE8)[2]. Potentially shielded H–bonds are highlighted with dashed lines.

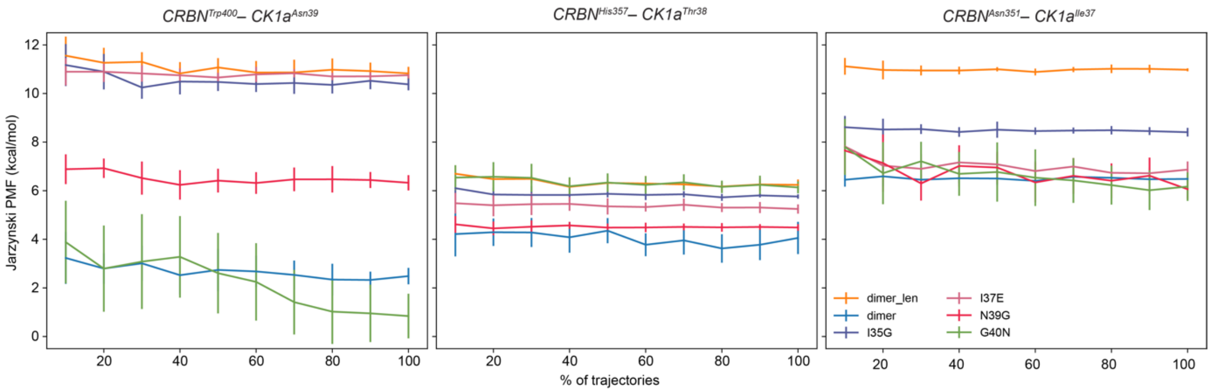

Figure S9: Sampling convergence of endpoint values of PMF_{HB_break} obtained using Steered Molecular Dynamics and the Jarzynski relationship. Error bars were obtained by bootstrapping 10 times the corresponding percentage of the total W profiles.

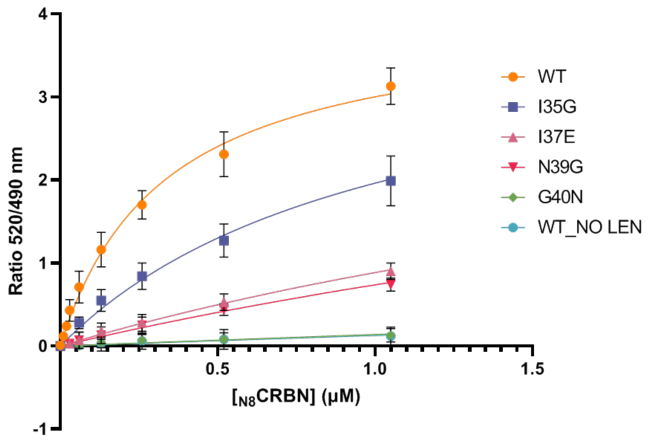

Figure S10: Dose-response plot from the 520/490 ratio obtained from Petzold et. al.[3] **(Supplementary Table S1) for all the CK1α mutants tested.** K_D values determined from this plot are listed in Supplementary Table 2

Table S1: Data values of the 520/490 ratio an estimated SD for the different single mutations of the CK1α as reported in Petzold et. al.[3] Numerical values were extracted with WebPlotDigitizer v 4.5[4]

[$_{N8}$CRBN] (μM)	^{wt}CK1α		^{wt}CK1α No LEN		^{I35G}CK1α		^{I37E}CK1α		^{N39G}CK1α		^{G40N}CK1α	
	**Ratio**	**SD**	**Ratio**	**SD**	**Ratio**	**SD**	**Ratio**	**SD**	**Ratio**	**SD**	**Ratio**	**SD**
1.05	3.13	0.22	0.12	0.11	1.99	0.30	0.90	0.10	0.74	0.08	0.13	0.08
0.52	2.31	0.27	0.08	0.12	1.27	0.20	0.53	0.10	0.44	0.07	0.08	0.08
0.26	1.70	0.17	0.06	0.10	0.84	0.16	0.28	0.10	0.25	0.10	0.07	0.07
0.13	1.16	0.21	0.03	0.09	0.55	0.13	0.18	0.10	0.13	0.10	0.04	0.06
0.06	0.71	0.19	0.01	0.00	0.28	0.07	0.07	0.10	0.07	0.10	0.01	0.00
0.03	0.43	0.13	N.D.	N.D.	N.D.	N.D.	0.03	0.00	0.03	0.00	N.D.	N.D.
0.02	0.24	0.00	N.D.	N.D.	N.D.	N.D.	N.D.	N.D.	N.D.	N.D.	N.D.	N.D.
0.01	0.12	0.00	N.D.	N.D.	N.D.	N.D.	N.D.	N.D.	N.D.	N.D.	N.D.	N.D.
0.00	0.01	0.00	0.00	0.00	0.00	0.00	0.00	0.00	0.00	0.00	0.00	0.00

Table S2: K_D values determined from the data values reported on Table S1. Fitting details provided in the Methods section.

	K_D (μM)	R squared
**^{wt}CK1α**	0.33 ± 0.03	0.9948
**^{wt}CK1α No LEN**	30.09 ± 8.48	0.8850
**^{I35G}CK1α**	1.04 ± 0.11	0.9920
**^{I37E}CK1α**	3.52 ± 0.18	0.9968
**^{N39G}CK1α**	4.43 ± 0.29	0.9944
**^{G40N}CK1α**	27.87 ± 9.24	0.8443

Other additional material

Dataset S1: An open data repository is accessible at https://bitbucket.org/jjuarez84/crbn-ck1a/src/master/

It contains:

- Topologies and equilibrated structures for each of the SMD systems.
- Sample amber input files and accessory files to perform the SMD protocol.
- Unprocessed W profiles obtained from SMD trajectories that were used to build the PMF_{HB_break} profiles.
- PDB files of the output of the MD simulations described in the manuscript
- **Movie S1: Selected SMD trajectories and associated W profiles.** SMD trajectories consistently show that the obtained W profiles are lower when water molecules can access the stretching H-bond.

Supplementary references

1. Anders, C. *et al.* A semisynthetic fusicoccane stabilizes a protein-protein interaction and enhances the expression of K+ channels at the cell surface. *Chem Biol* **20**, (2013).
2. Cao, S. *et al.* Defining molecular glues with a dual-nanobody cannabidiol sensor. *Nat Commun* **13**, 1–14 (2022).
3. Petzold, G., Fischer, E. S. & Thomä, N. H. Structural basis of lenalidomide-induced CK1α degradation by the CRL4 CRBN ubiquitin ligase. *Nature* **532**, 127–130 (2016).
4. Rohatgi, A. WebPlotDigitizer version 4.4. *Pacifica, California, USA* (2020).
